# Insight into Various Casting Material Selections in Rapid Investment Casting for Making EDM Electrodes

**DOI:** 10.3390/mi16050595

**Published:** 2025-05-20

**Authors:** Thanh Tan Nguyen, Van-Thuc Nguyen, Van Tron Tran, Anh Thi Le, Thanh Duy Nguyen, Quoc Dung Huynh, Minh Tri Ho, Minh Phung Dang, Hieu Giang Le, Van Thanh Tien Nguyen

**Affiliations:** 1 Faculty of Mechanical Engineering, Ho Chi Minh City University of Technology and Education, Ho Chi Minh City 71307, Vietnam or tannt25.ncs@hcmute.edu.vn (T.T.N.); gianglh@hcmute.edu.vn (H.G.L.); 2 Institute for Nanotechnology, Ho Chi Minh City 71308, Vietnam; 3 Vietnam National University Ho Chi Minh City, Ho Chi Minh City 71308, Vietnam; 4 Faculty of Mechanical Engineering, Industrial University of Ho Chi Minh City, Nguyen Van Bao Street, Ward 4, Go Vap District, Ho Chi Minh City 70000, Vietnam

**Keywords:** rapid prototyping, microstructures, shell thickness, shrinkage, surface roughness, crack

## Abstract

Investment casting is a precision casting technology that can produce complex shapes from various materials, particularly difficult-to-cast and difficult-to-machine metallic alloys. Meanwhile, electrical discharge machining (EDM) is a well-known technique for producing ultra-precise mechanical parts, and electrode quality is crucial. Few studies have explored how rapid prototyping (RP) pattern generation and investment casting influence the final product’s shape, dimensions, and surface roughness. This study investigates EDM electrode fabrication using investment casting and RP-generated epoxy resin patterns. We examine the effects of electrode materials (CuZn5, CuZn30, and FeCr24) on surface roughness, alongside the impact of ceramic shell thickness and RP pattern shrinkage on electrode quality. The EDM electrodes have a shrinkage of 0.8–1.9% and a surface roughness of 3.20–6.35 μm, depending on the material selections. Additionally, the probability of shell cracking decreases with increasing shell thickness, achieving stability at 16.00 mm. This research also applies investment casting electrodes to process DC53 steel. The results indicate that the surface roughness of the workpiece after EDM machining with different electrode materials is in the range of 4.71 µm to 9.88 µm. The result expands the use of investment casting in electrode fabrication, enabling the production of high-precision electrodes with complex profiles and challenging materials, potentially reducing both time and cost.

## 1. Introduction

There is an increasing desire for more complicated goods with shorter development cycles to attain lower total costs and higher quality. Modern molds exhibit increasingly complex geometries and employ high-hardness materials, challenging traditional machining. Electrical discharge machining (EDM) might solve the problem by using electrical discharge as a machining tool. About 50% of the die-sinking EDM process is spent producing rough-machining electrodes, which is the most time-consuming step. This is especially true if the electrode has a complicated appearance [1,2,3]. Therefore, optimizing this process could save a significant amount of time and expense. Rapid prototyping (RP) approaches are the cutting-edge method in the mold fabrication area. RP techniques can mainly produce EDM electrodes with complex shapes.

Numerous studies demonstrate that investment casting (IC) offers significant advantages for EDM electrode fabrication, enabling complex shapes, shorter lead times, and lower production costs [4]. This process allows for the fabrication of complex metal and alloy components, from single prototypes to high-volume production [5,6,7,8]. Moreover, the EDM technique can produce castings with a smooth surface finish, thin-walled structures, and complicated geometries [7]. Wax pattern molds for lost-wax casting are often created using classic machining techniques, such as turning, milling, and CNC machining. Tooling has limits such as minimum wall thickness, avoidance of abrupt radii, and cuts requiring larger draft angles, which result in higher production costs [8]. Traditional tooling for wax pattern molds is time-consuming and costly [9].

Several studies have explored 3D printing for investment casting and EDM electrode production [10,11]. Choudhari et al. investigated rapid 3D prototyping techniques [12]. Bansode et al. optimized electrode pattern design and assessed fracture susceptibility in investment casting [13]. Nguyen et al. and Choo et al. demonstrated that casting surface quality depends on 3D printing surface quality, with SLA 3D printing providing excellent resolution [14,15]. SLA can print high-fidelity patterns with resolutions up to 10 μm [16,17]. Kanyo et al. and Chen et al. reported that the creation of shell molds utilizing 3D printed materials can result in shell mold cracking, and they also provided strategies to prevent cracking using ceramic shell reinforcing [18,19]. Jones et al. [20] found that adding 20 µm diameter and 1 mm long nylon fibers to the ceramic layer improves shell strength and reduces the number of coating layers. Printing RP patterns could provide an interior hollow structure [11,21]. Idris et al. [22] identified that hollow, thin-walled patterns are more viable in investment casting. Material modifications to the pattern have been implemented to minimize the thermal expansion coefficient. According to Wang et al. [21], choosing a resin for investment casting requires careful consideration of the glass transition temperature, thermal expansion coefficient, and Young’s modulus. Furthermore, electrode materials must possess high levels of thermal conductivity, melting point, electrical conductivity, thermal conductivity, strong abrasion resistance, and high strength [23,24]. EDM electrodes are commonly made from materials such as copper, graphite, tungsten, brass, and silver. Nafi and Jahan [25] and Świercz et al. [26] show that the presence of oxides and carbides on the recast layer tends to increase surface micro-hardness. This can offer benefits in terms of wear resistance, potentially extending the electrode’s life. Similarly, carbon deposition on the copper anode surface can act as an additional thermal barrier, reducing electrode wear. On the other hand, for graphite electrodes, uneven carbon deposition can lead to unstable discharges, consequently increasing tool wear. This highlights the importance of the deposit’s uniformity. Furthermore, an undesirable or poorly structured recast layer can increase the surface roughness of the workpiece and potentially reduce effective machining speed. Recently, W-Ag, copper–titanium, and titanium have emerged as newer electrode materials in recent studies. These electrode materials offer significant improvements in durability, wear resistance, corrosion resistance, and machining efficiency compared to traditional materials in specific EDM applications [27,28,29,30].

Previous research has not sufficiently explained how the RP pattern generation and investment casting affect the final product’s shape, dimensions, and surface roughness. Furthermore, research on various alloys for investment casting EDM electrodes has been relatively limited. FeCr24 is an excellent wear-resistant, thermally stable, and hard alloy adaptable to casting processes. To address existing problems, this study investigates how ceramic shell thickness influences the creation of the shell mold, the surface roughness relation of the 3D printed pattern, the shell mold, and the CuZn5, CuZn30, and FeCr24 alloy electrodes on investment casting. This study also compares the workpiece surface roughness obtained from those three alloys. This work highlights the practicability of producing EDM electrodes using investment casting with 3D-printed patterns. The findings of this study could expand the use of investment casting in electrode fabrication and enable the production of high-precision electrodes with complex profiles and challenging materials, potentially reducing both time and cost.

## 2. Materials and Methods

### 2.1. Materials

#### 2.1.1. Electrode Design

**Figure 1:**
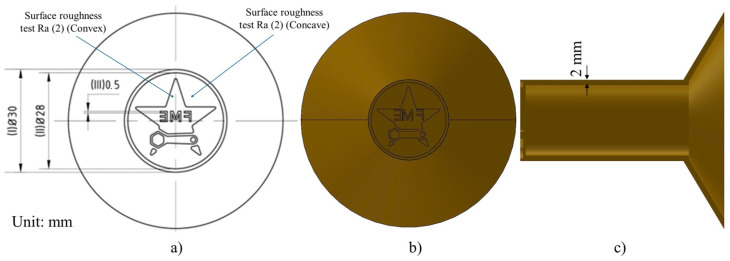
Electrode model: (**a**) 2D design, (**b**,**c**) front and side view of the electrode.

The EDM electrode pattern model was created with Creo Parametric 8.0 software from Parametric Technology Corporation (Boston, MA, USA). This pattern model employed a hollow, thin-sectioned design. The geometry and dimensions of the electrode are illustrated in Figure 1.

#### 2.1.2. Materials Properties

Table 1 shows the chemical composition and physical characteristics of the electrode material after casting, using the optical emission spectrometry (OES) analyzer SPECTROMAXx (SPECTRO Analytical Instruments GmbH, Boschstrasse, Kleve, Germany). The chemical composition of the material is comparable to CuZn5, CuZn30, and FeCr24, respectively. The material’s physical properties are presented in Table 2.

### 2.2. Methods

#### 2.2.1. SLA 3D Fabrication Process of Electrodes

**Figure 2:**
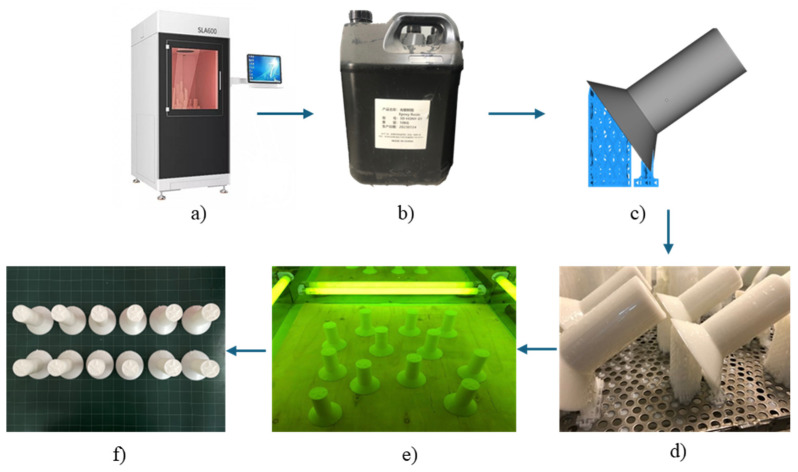
Prototyping process using SLA 3D printing method: (**a**) Zongheng SLA-600 3D printer, (**b**) epoxy HONY-01 resin, (**c**) pattern support structures, (**d**) pattern during the 3D printing process, (**e**) UV curing, and (**f**) final 3D printed pattern.

Figure 2 shows the electrode pattern production process using 3D SLA printing. After 3D design, the file was converted to the .stl file format. Then, the electrode patterns were printed using a Zongheng SLA-600 printer (ZONGHENG3D company, Xiangzhou District, Zhuhai, China) with ultraviolet rays, a wavelength of 355 nm, and a power source of 3 W, as illustrated in Figure 2a. The electrode patterns were printed with a layer resolution of 0.1 mm, the 3D part inclined at 45° with support, and a pattern thickness of 2.0 mm, using epoxy resin HONY-01 (Zongheng Additive Intelligent Technology Co., Ltd., Zhuhai, China), as shown in Figure 2b. The printing parameters and orientation were configured to ensure a distinct logo surface and minimal roughness, as shown in Figure 2c. The patterns were fabricated under identical conditions and in a single print batch, as shown in Figure 2d. After printing, as shown in Figure 2e, the printed electrode patterns underwent UV curing to achieve complete resin solidification. Figure 2f shows the final 3D-printed electrodes used for the investment casting process.

#### 2.2.2. Electrode Fabrication Process via Lost-Wax Casting

**Figure 3:**
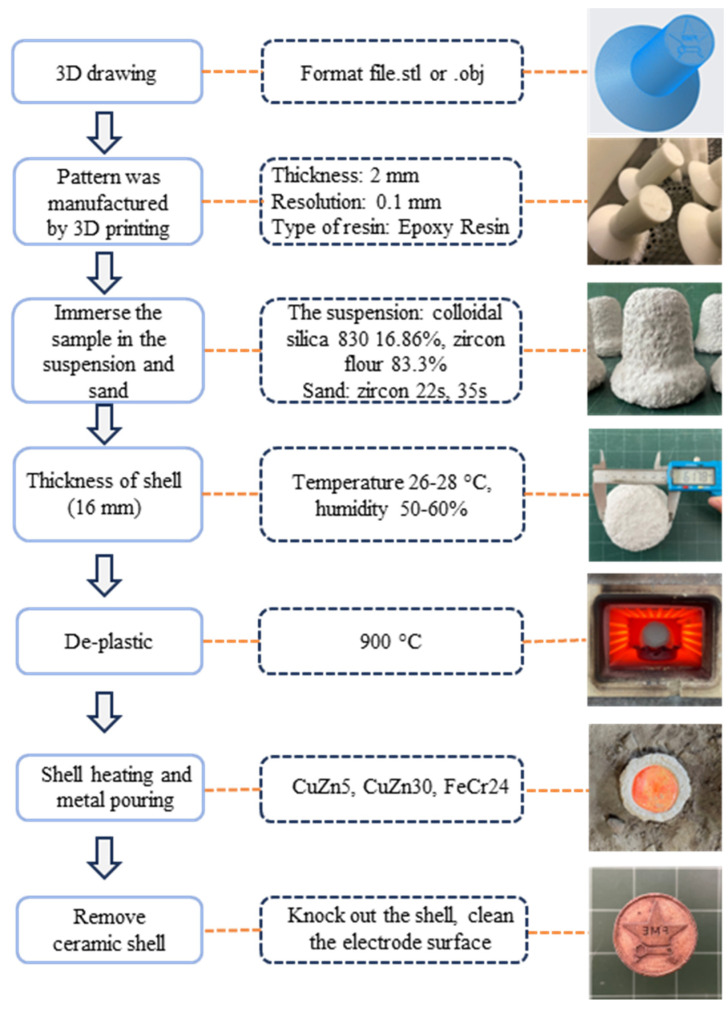
The fabrication process of EDM electrodes from 3D printing patterns.

The fabrication of electrodes utilizing the investment casting method was performed in a sequence of steps, as illustrated in Figure 3. Initially, the 3D printed pattern was immersed in a slurry composed of 18.67% silica 830 and 83.33% zircon flour, followed by the application of a 22–35s sand coating and subsequent drying at a temperature range of 26–28 °C and a humidity range of 50–60% for 24 h. The immersion and coating technique was continued until the mold shell reached the appropriate thickness. The polymer was then degraded, and the mold was sintered at 900 °C to form the ceramic shell. Next, CuZn5, CuZn30, and FeCr24 alloys were poured into the mold. Finally, the shell was destroyed, and the surface was cleaned to reveal the final electrodes.

#### 2.2.3. Machining Process and Measurement Equipment

**Figure 4:**
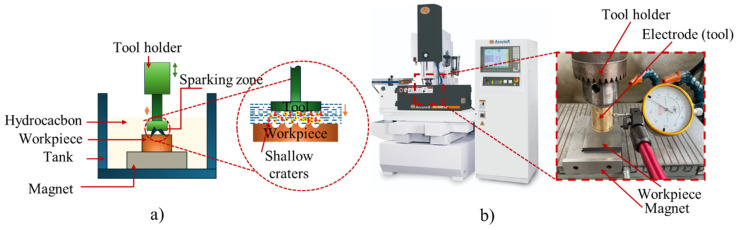
EDM machine: (**a**) EDM principle, (**b**) AccuteX DS-430S CM EDM machine.

Figure 4a depicts the fundamental principle of electrical discharge machining (EDM). The electrode was fixed to Taiwan’s AccuteX DS-430S CM machine (Nantun District, Taichung City, Taiwan) (Figure 4b). The machining was performed using S1 code in the machine, with its parameters and technical specifications detailed in Table 3. EDM machining uses a dielectric fluid (APIG EDM FLUID, Hao Ky Ltd., Ho Chi Minh City, Vietnam), which is typically a hydrocarbon, and the workpiece is submerged in the dielectric solution.

**Figure 5:**
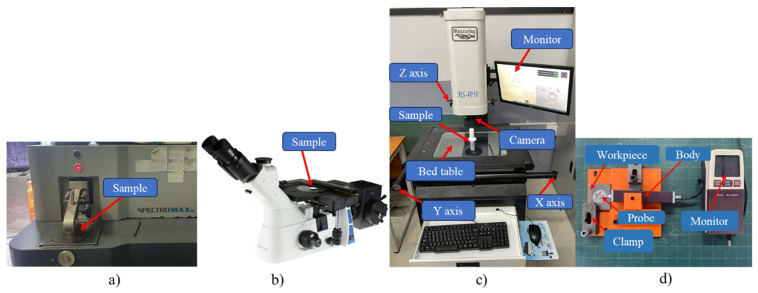
Equipment for the experiment: (**a**) OES analyzer SPECTROMAXx, (**b**) Euromex OX.2653-PLM microscope, (**c**) manual vision measuring machine MS-4030 VMM, (**d**) roughness tester Mitutoyo SJ-201.

The equipment for the experiment is shown in Figure 5. The chemical composition of the casted electrode was checked by SPECTROMAXX LMX10 (SPECTRO Analytical Instruments GmbH, Boschstrasse, Kleve, Germany) (Figure 5a). The microstructure analysis of the 3D-printed patterns and electrodes was conducted using a Euromex OX.2653-PLM microscope, Euromex Microscope B.V (VB Duiven, The Netherlands) (Figure 5b). A Manual vision measuring machine named VMM MS-4030 (Xinzhuang, New Taipei City, Taiwan) was employed to determine the surface geometry dimensions of the electrodes (Figure 5c). The surface roughness was quantified using a Mitutoyo SJ-201 roughness measurement instrument in Mitutoyo (Kawasaki, Kanagawa, Japan) (Figure 5d) [32,33]. The test is measured at 2 positions, convex (Ra (1)) and concave (Ra (2)) (Figure 1).

The following formula determines the shrinkage of the product:

(1) $$\%\;Shrinkage=[1-\left(\frac{N_{ave}}{Nominal\;dimension}\right)]\times 100$$

where *N_ave_* is the average of 3 measurements, which is calculated by:

(2) $$N_{ave}=\frac{N1+N2+N3}{3}$$

where N1, N2, and N3 are the dimensions of the product.

## 3. Results and Discussion

### 3.1. Effect of Mold Shell Thickness on Mold Durability

In the IC process, the composition and thickness of the mold shell should ensure the casting’s roughness and dimension accuracy and prevent cracking during the dewaxing and metal casting stages. This study investigated the effect of mold shell thickness on the fracture phenomenon. Table 4 indicates the thickness of the mold shell after coatings.

**Figure 6:**
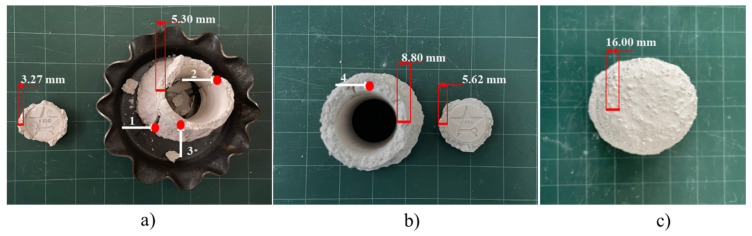
Cracking patterns in different ceramic shells: (**a**) 5.3 mm shell thickness (4 layers), (**b**) 8.8 mm shell thickness (6 layers), and (**c**) 16.0 mm shell thickness (10 layers).

After ceramic coating, the patterns underwent a degradation phase in a furnace, with temperature gradients ranging from 30 to 900 °C. In the case of the 5.3 mm thickness, a fracture was observed at 200 °C during the degradation phase. The ceramic shell, which was 8.80 mm thick, also exhibited cracking at 400 °C. The observed fractures are attributed to stress concentration at the corners and edges of the ceramic shell, as evidenced in Points 1, 2, and 3 in Figure 6a and Point 4 in Figure 6b. Notably, the 16.0 mm thick ceramic shell remained intact throughout the degradation process, as depicted in Figure 6c. The fracture regions in the 5.3 mm thick shell are measured at 3.27 mm, while those in the 8.8 mm thick shell are measured at 5.62 mm, indicating a thickness reduction of 36.1–38.3% compared to the non-fractured regions. The reduced surface area decreases volume density at the corners/edges, thereby exacerbating crack propagation at stress concentration [18,29]. Furthermore, considerable differences in the coefficient of thermal expansion (CTE) between the pattern material (epoxy resin) and the ceramic shell (SiO_2_) are also the reason for the fracture. SiO_2_ has a CTE of 0.6 × 10^−6^ K^−1^, while epoxy resin has a CTE of 59.9 × 10^−6^ K^−1^, almost 200 times higher [21,22,34]. The CTE mismatch between pattern and shell materials induces stress exceeding the shell’s fracture modulus [19,20,21,22]. Experimentally, increased shell thickness demonstrably enhances fracture resistance [13]. Besides increasing the thickness of the shell to enhance mold strength, the mold’s durability can also be improved by altering the material composition, adding reinforcing fibers, etc. [35,36].

### 3.2. Evaluation of Shrinkage in the Electrode Fabrication Process

**Figure 7:**
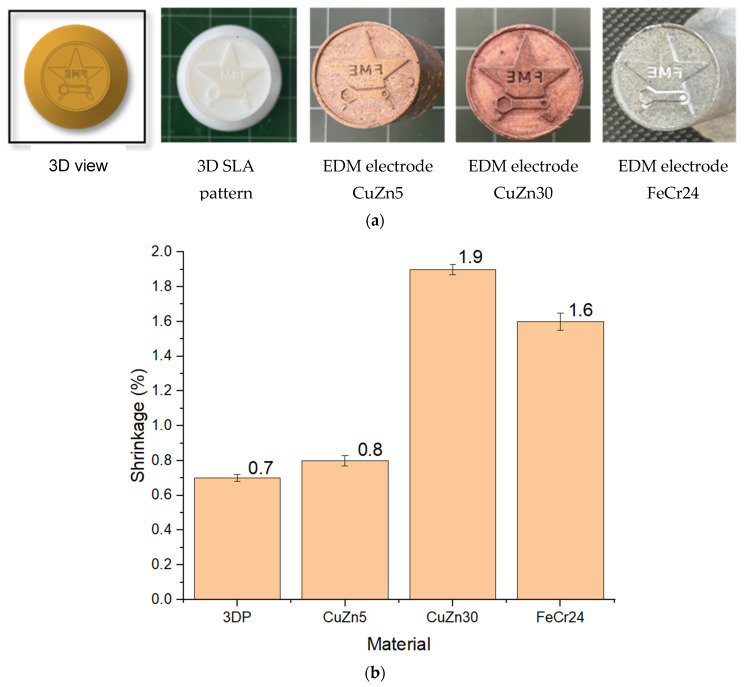
EDM electrodes from the IC process include (**a**) 3D view, 3D SLA pattern, EDM electrode from CuZn5, CuZn30, and FeCr24, and (**b**) shrinkage of the RP and the IC electrodes.

Figure 7a shows the electrode model, 3D SLA pattern, and the IC electrodes from CuZn5, CuZn30, and FeCr24. The dimension shrinkage of the 3D-printed patterns and cast specimens is illustrated in Figure 7b. The results reveal that the dimensional shrinkage of the 3DP patterns, CuZn5, CuZn30, and FeCr24, is 0.7%, 0.8%, 1.9%, and 1.6%, respectively. The RP pattern dimensions are consistently smaller than the nominal dimensions, within a range of 0.7%. The disparity between the RP pattern and nominal dimensions is attributed to the inherent shrinkage of the epoxy resin material during the 3D SLA printing process [37,38,39]. This result is consistent with prior research, which has reported shrinkage values ranging from 0.008 to 3.67% [11] and 0.183 to 0.50% [12]. The reason is the resolution limitations of the continuously stacked material layers, leading to dimensional variations (the printing resolution of the Zongheng SLA-600 apparatus is 0.1 mm). Moreover, phase transformations such as gelation, vitrification, and layer-by-layer solidification’s cumulative effect over time result in volumetric shrinkage [40]. Thermal fluctuations during 3D printing also contribute to volumetric shrinkage [39]. This contraction requires pre-compensation to assure the accuracy of the final IC electrodes. Besides 3DP pattern shrinkage, the shrinkage value also presents alloy shrinkage during solidification [41]. The castings exhibit shrinkage compared to the initial nominal dimensions due to metals contracting when solidified, reducing volume. Moreover, different casting methods could also lead to different shrinkage rates [42,43,44]. Specifically, CuZn5 shrinks by 0.8%, CuZn30 shrinks by 1.9%, and FeCr24 shrinks by 1.6%. Because the freezing range of CuZn5 is shorter than CuZn30 [45,46]. This shrinkage result is consistent with the Machuta et al. study [47].

**Figure 8:**
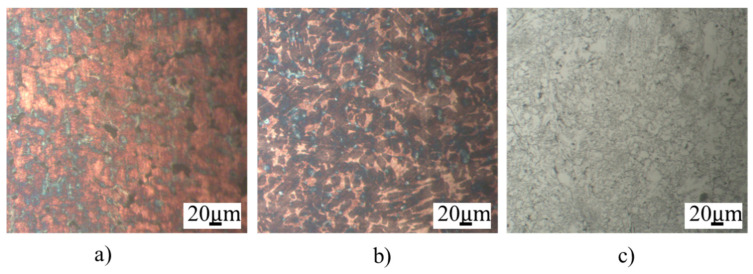
Microstructure of different IC electrodes: (**a**) CuZn5, (**b**) CuZn30, and (**c**) FeCr24.

The microstructures of the IC electrodes are shown in Figure 8. The microstructures of CuZn5 and CuZn30 present the single-phase structure of the α phase. The microstructure of FeCr24 is composed mainly of the ferrite phase and some carbides with dark color scattering on the ferrite matrix. Interestingly, the grain size of the FeCr24 sample is finer than the CuZn5 and CuZn30 samples.

### 3.3. Evaluation of Surface Roughness of RP Patterns, Electrodes, and Workpiece After EDM Machining

**Figure 9:**
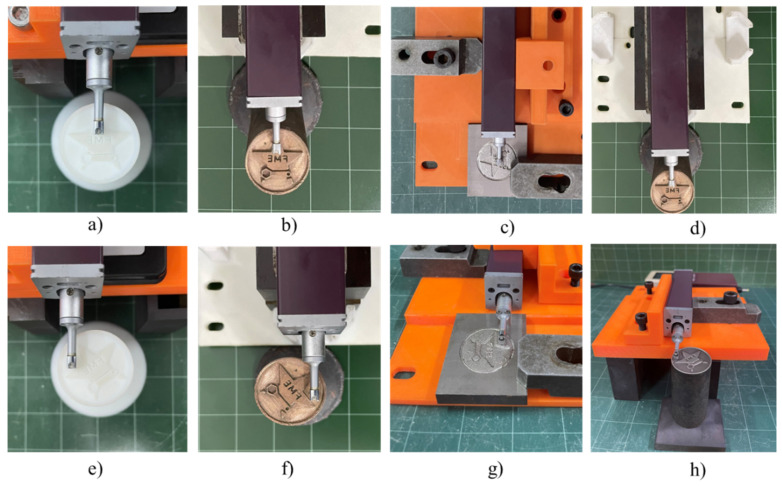
Surface roughness test: (**a**) 3DP model, (**b**) cast specimen, (**c**) EDM machined workpiece, and (**d**) EDM machined electrode at the Ra (1) position; (**e**) correspond to the 3DP model, (**f**) cast specimen, (**g**) EDM machined workpiece, and (**h**) EDM machined electrode at Ra (2) position.

**Figure 10:**
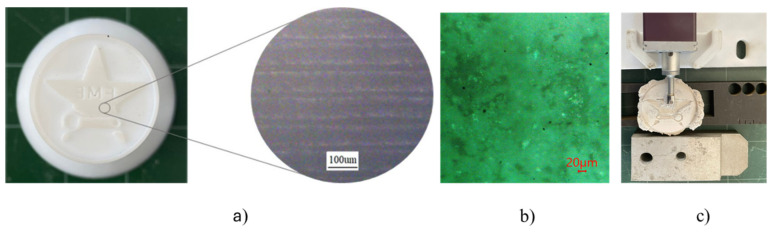
The stair-step effect in AM: (**a**) surface of the 3DP with stair-step, (**b**) shell mold microstructure, and (**c**) roughness test of the ceramic shell.

**Figure 11:**
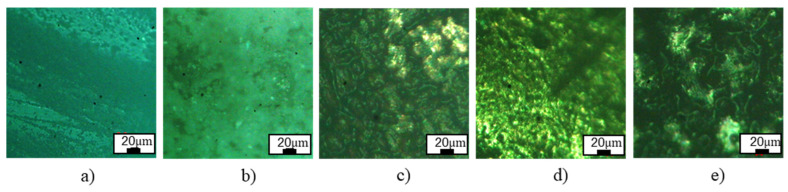
Microscopic inspection of the sample surface: (**a**) 3D pattern, (**b**) shell mold, (**c**) CuZn5 electrode, (**d**) CuZn30 electrode, (**e**) FeCr24 electrode.

**Figure 12:**
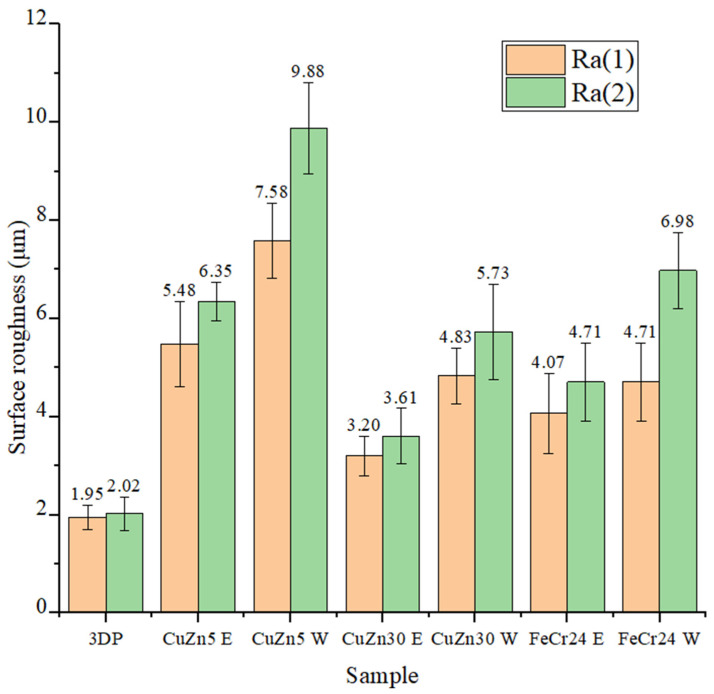
After EDM machining with different material types, the surface roughness distribution chart of 3D-printed patterns, electrodes, and workpiece (3DP: 3D-printed pattern, E: electrode, W: workpiece, Ra1: first position, Ra2: second position).

The surface roughness of the 3DP model cast specimens, machined workpiece post-EDM, and electrodes post-EDM was assessed at two distinct locations, as shown in Figure 9. Figure 10 shows the stair-step effect in AM, while Figure 11 presents the microscopic inspection of the sample surface. The numerical analysis is summarized in Figure 12.

The reason for the surface roughness morphology is the “stair-stepping” effect. Yang et al. [48] also found that surface roughness finishing issues in 3D-printed items are caused mainly by the “stair-stepping” effect inherent in additive manufacturing (AM), as mentioned in Figure 10. This phenomenon results from layer-by-layer production and limits part precision and performance. Regarding shell mold surface roughness, Kumar et al. [49] proposed that ceramic slurry materials applied to RP models with smoother surfaces produce improved interior shell mold finishes; however, these materials reduce shell permeability. Shell permeability is crucial for air evacuation during molten metal casting, reducing porosity and porosity flaws while maintaining surface roughness [49]. These factors collectively contribute to the elevated final surface roughness observed in the electrodes.

Figure 12 depicts an analysis of surface roughness measurements. The cast specimens have higher surface roughness than the 3D-printed models. Moreover, the surface roughness of the EDM workpieces is always higher than that of the related electrode. The surface roughness varies between places, with position 2 having more roughness than location 1. The electrode roughness values at the Ra1 position are 5.48 µm, 3.2 µm, and 4.07 µm, corresponding to the electrode materials CuZn5, CuZn30, and FeCr24. The CuZn30 electrode has the lowest surface roughness, while the CuZn5 electrode has the highest. In addition, the surface finish of the investment cast product is determined by the surface finish of the 3D-printed model and the internal surface finish of the ceramic shell. The roughness of the material surfaces increases gradually from the 3D-printed model manufacturing to mold shell formation and investment casting.

**Figure 13:**
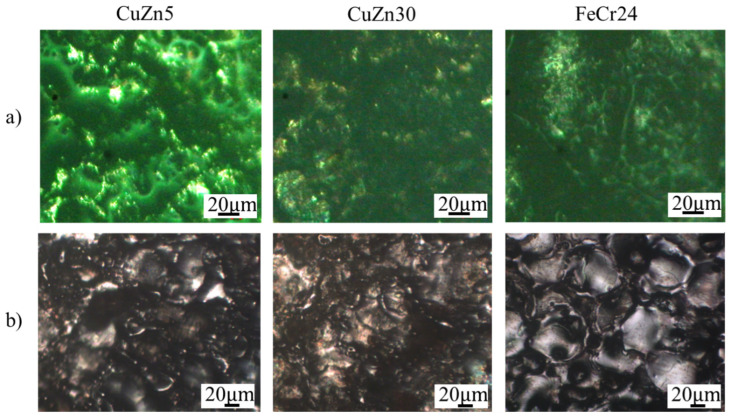
Surface morphology of electrode casting. (**a**) Surface morphology of the electrode after EDM machining. (**b**) Surface morphology of the workpiece after EDM machining.

Figure 13a depicts the electrode material’s surface morphology after machining, whereas Figure 13b depicts the surface morphology of the machined material. The examination shows that the machined material has a rougher surface than the electrode. This is due to the workpiece’s anodic connection during machining, which causes higher temperatures and increased ion bombardment, leading to greater surface roughness, consistent with other findings [50,51].

### 3.4. Comparison of the Electrode Wear Rate (EWR) Performance of Investment Casting Electrodes vs. CNC Electrodes and MRR, EWR, WSR of CuZn5, CuZn30, and FeCr24

**Figure 14:**
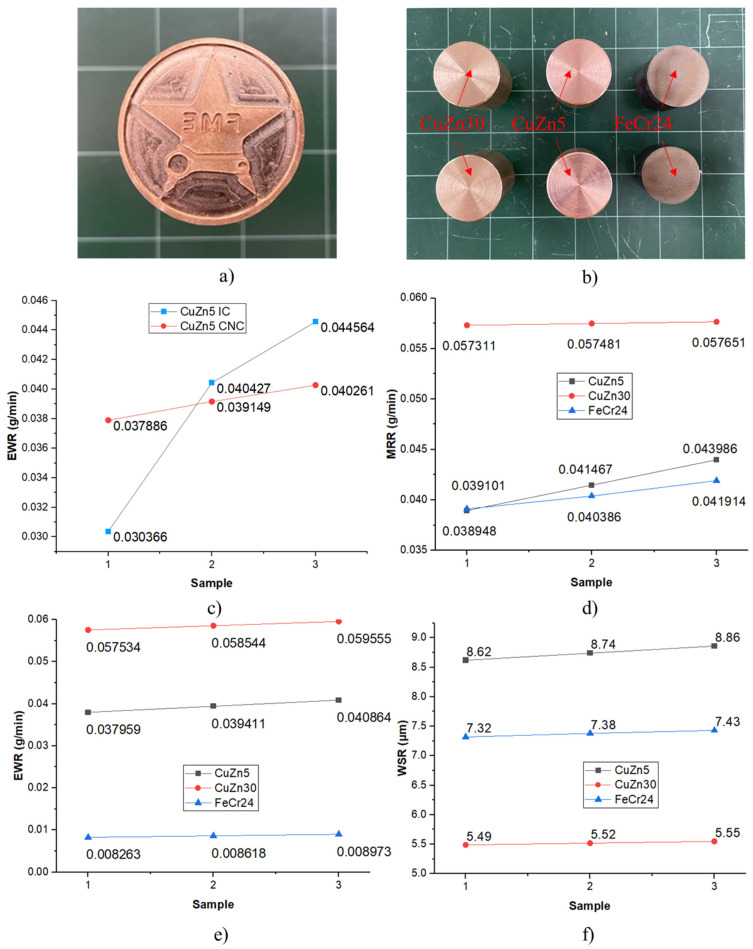
Comparison between different types of electrodes: (**a**) CNC electrode, (**b**) MRR comparison between IC electrode and CNC electrode, (**c**) IC electrodes, (**d**) MRR comparison, (**e**) EWR comparison, and (**f**) WSR comparison.

This section compares the EDM machining performance of the CNC electrode (Figure 14a) with the IC electrode, as well as between three IC electrode materials—CuZn5, CuZn30, and FeCr24 (Figure 14b). The electrodes have the same geometric profile (Φ30) and are machined with the same DC53 steel, parameters, and working conditions. The EDM machining parameter set includes I = 5 A, Ton = 120, and Toff = 60; other parameters are set using the S1 code in Table 3.

Figure 14a,c show the CNC electrode and the result of EWR for both electrode types, CuZn5 IC and CuZn5 CNC, during the EDM process under the same conditions (I = 5 A, Ton = 120 µs, Toff = 60 µs). As shown in Figure 14c, the EWR value of the CuZn5 IC is lower than that of CuZn5 CNC in the first sample and then higher. The difference in electrode wear rate, despite the electrodes being of the same CuZn5 material, is due to the initial surface roughness of the electrodes. Research by Hadad et al. [50] indicates that an electrode with a higher initial surface roughness will experience a greater electrode wear rate under the same EDM machining conditions. The CuZn5 CNC electrode, which has many material peaks, will quickly reach the maximum number of peaks and have an effective spark discharge area during EDM machining. In contrast, the CuZn5 IC electrode, due to its high surface roughness, will have a lower number of material peaks. However, the volume of these peaks is larger than the peak volume of the CuZn5 CNC electrode, so the time to reach the maximum number of peaks and an effective spark discharge area will be longer, resulting in a higher EWR compared to the CuZn5 CNC electrode.

Observing the graphs in Figure 14d,e, it is seen that the MRR of the CuZn30 electrode is the fastest at 0.057 g/min, followed by the CuZn5 electrode (0.041 g/min), and finally, the FeCr24 electrode (0.040 g/min). The result is consistent with the Mohanty et al. study, which presented a value of 0.01886195 g/min [3]. Figure 14e shows that the EWR of the CuZn30 electrode is the highest (0.058 g/min), followed by the CuZn5 electrode (0.039 g/min), and lastly, the FeCr24 electrode (0.088 g/min). The results are consistent with Mohanty et al.’s research, with 0.03549–0.08932 g/min [3]. Figure 14f demonstrates that the surface roughness of the DC53 workpiece machined by the CuZn5 electrode is the highest at 8.7 μm, followed by the FeCr24 electrode at 7.3 μm, and lastly, the CuZn30 electrode at 5.5 μm. This result is also in line with previous studies: 1.71–4.686 μm [2], SR = 3.14–5.133 μm [3], and 2.48–7.4 μm [52].

**Figure 15:**
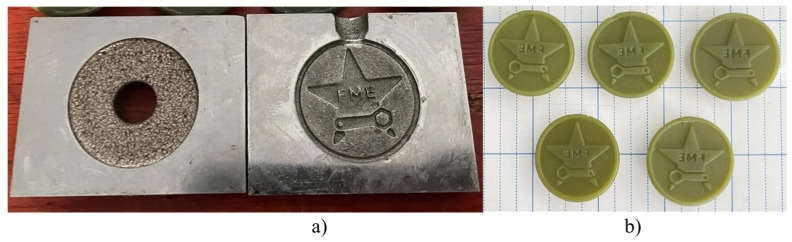
The application of an electrode to create the mold for wax pattern: (**a**) mold created from investment casting electrode, and (**b**) wax pattern from the mold.

Surprisingly, the study’s methodology is also used to create wax pattern molds for investment casting. Figure 15a shows the final machined mold, whereas Figure 15b shows the wax pattern generated using the investment cast electrode. Surface roughness in pattern molding ranges from 4.26 to 4.32 µm, comparable to the 3.25 to 3.68 µm value for CNC electrodes. This indicates that investment-cast electrodes can produce wax pattern molds for investment casting.

## 4. Conclusions

This study examines the effects of electrode materials on the surface roughness of product finishing for wax pattern mold in the IC process. Some critical conclusions are listed as follows:

- There is a clear correlation between ceramic shell thickness and fracture resistance during the degradation phase. Thinner shells were susceptible to cracking due to stress concentration and the substantial difference in CTE between the ceramic and the epoxy pattern. The ceramic shell thickness considerably impacts the product development process in investment casting. Increased thickness leads to higher durability. Increasing the shell thickness of up to 16.0 mm (10 layers) helps against the thermal expansion of 3D-printed designs. The patterns are created by SLA 3D printing with epoxy resin, with a dimensional shrinkage of 0.7% and a surface roughness of 1.95–2.02 μm.
- The shrinkage and surface finish of the investment cast electrode depend on the surface finish of the 3D-printed pattern, the internal surface finish of the ceramic shell, and the material selection. EDM-cast electrodes have a shrinkage of 0.8–1.9% and a surface roughness of 3.2–6.35 μm, depending on the materials chosen.
- The shrinkage and surface roughness of CuZn5, CuZn30, and FeCr24 electrodes are 0.8% and 5.48–6.35 µm, 1.9% and 3.20–3.61 µm, and 1.6% and 4.07–4.71 µm, respectively. The machined surface roughness of the workpiece using CuZn5, CuZn30, and FeCr24 electrodes is 7.58–9.88 µm, 4.83–5.73 µm, and 4.71–6.98 µm, respectively.
- Investment casting allows for fabricating post-cast products with complex geometries and high-hardness materials, such as FeCr24, with modest surface roughness. The roughness of the wax pattern created from the machined mold, applying the investment cast electrode, ranges from 4.26 to 4.32 µm, which is acceptable in rough machining. The 3D SLA printing technology in investment casting has shown significant potential for product development and tooling in the EDM technique. The electrode wear rate of as-cast EDM electrodes is slower in the initial stage compared to CNC-machined electrodes, and then it wears out faster.

## Figures and Tables

**Table 1:** Chemical compositions of rapid investment casting electrodes.

Materials	Chemical Composition (%)										
	Fe	Ni	Cu	Zn	Sn	Pb	Cr	C	Si	Mn	V
FeCr24	71.000	0.224	0.0402	0.005	0.003	0.002	24.130	2.990	0.835	0.447	0.119
CuZn30	1.230	0.265	69.930	26.640	1.000	0.918	-	-	-	-	-
CuZn5	0.094	0.473	85.730	4.720	4.340	4.640	-	-	-	-	-

**Table 2:** Physical properties of CuZn5, CuZn30, and FeCr24 [31].

Materials	CuZn5	CuZn30	FeCr24
Melting temperature ℃	1030	954	1150
Electrical conductivity MS/m	8.5	16	0.91
Thermal conductivity W/mK	71	121	17

**Table 3:** Basic parameters of the AccuteX DS-430S CM EDM machine.

Parameters	Values
X-axis	400 mm
Y-axis	300 mm
Z-axis (Servo)	300 mm
Table working	650 × 350 mm
Spacing from the chuck to the machine table	150–450 mm
HV (High Voltage)	1A (V)
GAP (Distance between electrode and workpiece)	9
SERVO	62.5%
JI (Jump backward distance)	2 (mm)
WT (Duration of sparking)	0.3 (s)

**Table 4:** Thickness of the mold shell (mm).

Mold Types	Samples	Measurements			Average
		1	2	3	
4 layers (5.3 mm)	A	5.29	5.43	5.25	5.32
	B	5.26	5.44	5.16	5.29
	C	5.37	5.29	5.43	5.36
6 layers (8.8 mm)	A	8.76	8.78	8.81	8.78
	B	8.75	8.66	8.53	8.64
	C	8.71	9.08	9.12	8.97
6 layers (16.0 mm)	A	16.02	16.29	16.22	16.18
	B	16.29	16.08	15.99	16.12
	C	15.94	16.20	16.02	16.05

## Data Availability

The datasets used and/or analyzed during the current study are available from the corresponding author upon reasonable request.
